# Whole genome sequencing of OXA-232-producing *wzi93*-KL112-O1 carbapenem-resistant *Klebsiella pneumoniae* in human bloodstream infection co-harboring chromosomal IS*Ecp1*-based *bla*_CTX-M-15_ and one *rmpA2*-associated virulence plasmid

**DOI:** 10.3389/fcimb.2022.984479

**Published:** 2022-09-29

**Authors:** Chongmei Tian, Mengyu Xing, Yaping Zhao, Xueyu Fan, Yongfeng Bai, Liping Fu, Siwei Wang

**Affiliations:** ^1^ Department of Pharmacy, Shaoxing Hospital of Traditional Chinese Medicine Affiliated to Zhejiang Chinese Medical University, Shaoxing, China; ^2^ Department of Pharmacy, Affiliated Hangzhou First People’s Hospital, Zhejiang University School of Medicine, Hangzhou, China; ^3^ Department of Clinical Laboratory, The Quzhou Affiliated Hospital of Wenzhou Medical University, Quzhou People’s Hospital, Quzhou, China; ^4^ Core Facility, The Quzhou Affiliated Hospital of Wenzhou Medical University, Quzhou People’s Hospital, Quzhou, China

**Keywords:** CRKP, OXA-232, chromosomal *bla*_CTX-M-15_, pLVPK-like virulence plasmid, IS*26*, co-integration

## Abstract

**Objectives:**

To characterize one OXA-232-producing *wzi93*-KL112-O1 carbapenem-resistant *Klebsiella pneumoniae* (CRKP) co-harboring chromosomal *bla*_CTX-M-15_ and one *rmpA2*-associated virulence plasmid.

**Methods:**

Minimum inhibitory concentrations (MICs) were measured *via* broth microdilution method. Conjugation, chemical transformation, string test and *Galleria mellonella* infection model experiments were also conducted. Whole-genome sequencing (WGS) was performed on the Illumina and Nanopore platforms. Antimicrobial resistance determinants were identified using ABRicate program with ResFinder database. Insertion sequences (ISs) were identified using ISfinder. Bacterial virulence factors were identified using virulence factor database (VFDB). Wzi, capsular polysaccharide (KL) and lipoolygosaccharide (OCL) were analyzed using Kleborate with *Kaptive*. Phylogenetic analysis of 109 ST15 *K. pneumoniae* strains was performed using core genome multilocus sequence typing (cgMLST) on the Ridom SeqSphere+ server. MLST, replicons type, SNP strategies and another cgMLST analysis for 45 OXA-232-producing *K. pneumoniae* strains were further conducted using BacWGSTdb server.

**Results:**

*K. pneumoniae* KPTCM strain belongs to ST15 with *wzi93*, KL112 and O1. It possessed a multidrug-resistant (MDR) profile and was resistant to carbapenems (meropenem and ertapenem), ciprofloxacin and amikacin. Virulence assays demonstrated KPTCM strain possesses a low virulence phenotype. WGS revealed it contained one circular chromosome and nine plasmids. The carbapenemase-encoding gene *bla*_OXA-232_ was located in a 6141-bp ColKP3-type non-conjugative plasmid and flanked by ΔIS*Ecp1* and Δ*lysR*-Δ*ereA*. Interestingly, *bla*_CTX-M-15_ was located in the chromosome mediated by IS*Ecp1-*based transposon Tn*2012*. Importantly, it harbored a *rmpA2*-associated pLVPK-like virulence plasmid with *iutA*-*iucABCD* gene cluster and one IS*26*-mediated MDR fusion plasmid according to 8-bp (AGCTGCAC or GGCCTTTG) target site duplications (TSD). Based on the cgMLST and SNP analysis, data showed OXA-232-producing ST15 *K. pneumoniae* isolates were mainly isolated from China and have evolved in recent years.

**Conclusions:**

Early detection of CRKP strains carrying chromosomal *bla*_CTX-M-15_, OXA-232 carbapenemase and pLVPK-like virulence plasmid is recommended to avoid the extensive spread of this high-risk clone.

## Introduction

*Klebsiella pneumoniae* is an important opportunistic pathogen associated with hospital- and community-acquired infections that has gained public attention due to its capability of acquiring resistance genes and plasmids (Kong et al., 2021a; Xu et al., 2021). Specifically, the emergence of carbapenem-resistant *K. pneumoniae* (CRKP) has limited effective therapies and poses a tremendous challenge in clinical settings (He et al., 2022). It is well documented that *K. pneumoniae* usually is classified into classical *K. pneumoniae* (cKP) and hypervirulent *K. pneumoniae* (hvKP) based on different virulence levels (Tian et al., 2021). In general, CRKP belong to the cKP group. When compared with cKP, hvKP mainly causes bloodstream infections (BSIs) and liver abscesses, both of which pose huge threat to patients (Wang et al., 2018; Wanford et al., 2021). Hypervirulent phenotype is mainly relevant to *rmpA*/*rmpA*2, aerobactin (*iucABCD* and *iutA*) and salmochelin (*iroBCDN*). These virulence factors are usually on a pLVPK-like virulence plasmid. (Kong et al., 2021b). Of greater concern, the amount of carbapenem-resistant hypervirulent *K. pneumoniae* (CR-hvKP) is increasing and infections caused by CR-hvKP strains generally lead to severe outcomes and mortality (Gu et al., 2018; Chen et al., 2021a).

OXA-232, a type of OXA-48-like enzyme, differs from OXA-48 and OXA-181 by five and one amino acid substitutions, respectively (Potron et al., 2013; Lahlaoui et al., 2017). OXA-232 could hydrolyze temocillin, penicillins, cefotaxime, cefepime and carbapenems with different hydrolytic activities (Potron et al., 2013). Catalytic activities of OXA-232 for all penicillins except temocillin (ten-fold lower) were two- to six-fold higher than OXA-181 and OXA-48, respectively (Potron et al., 2013; Oueslati et al., 2015). The catalytic activity of OXA-232 for imipenem was quite lower than OXA-181 and OXA-48. Hydrolysis abilities of meropenem and ertapenem were similar among OXA-232, OXA-48 and OXA-181 (Potron et al., 2013).

OXA-232-producing *K. pneumoniae* strains have been reported in Zhejiang and Shanghai, China (Shen et al., 2020b; Shi et al., 2020; Jia et al., 2021), but to our knowledge, no comprehensive phylogenetic analysis for ST15 *K. pneumoniae* isolates collected from various countries has been performed before. Moreover, a study by Zhu *et al*. in Yancheng, China reported clonal dissemination of ST15 OXA-232-producing *K. pneumoniae* strains (Zhu et al., 2021). Based on the short-read Illumina whole-genome sequencing (WGS), *bla*_CTX-M-15_ was found in an IncFIIK-type plasmid (Zhu et al., 2021). In addition, there are no further corresponding description about *rmpA2*-related virulence plasmid structure and virulence assessment experiments in their ST15 OXA-232-producing *K. pneumoniae* strains.

Here, one OXA-232-producing sequence type (ST) 15 CRKP in human bloodstream infection was isolated in January 2022 in China and the complete genetic characteristics were further investigated. This study provides a comprehensive description of the complete genomic features of a *wzi93*-KL112-O1 OXA-232-producing ST15 CRKP, which harbors a *rmpA2*-related pLVPK-like virulence plasmid and the chromosomal *bla*_CTX-M-15_ mediated by IS*Ecp1*-based transposon Tn*2012*. Moreover, a multi-drug resistance plasmid may have evolved through IS*26*-mediated co-integration. This information will offer help to prevent and control the extensive spread of OXA-232-producing ST15 *K. pneumoniae* isolates. Importantly, the combination of short-read Illumina and long-read MinION WGS was performed to provide complete insight into the genomic structure features of resistance and virulence plasmids of *K. pneumoniae*.

## Materials and methods

### Flow chart illustrating the study process

**Figure 1:**
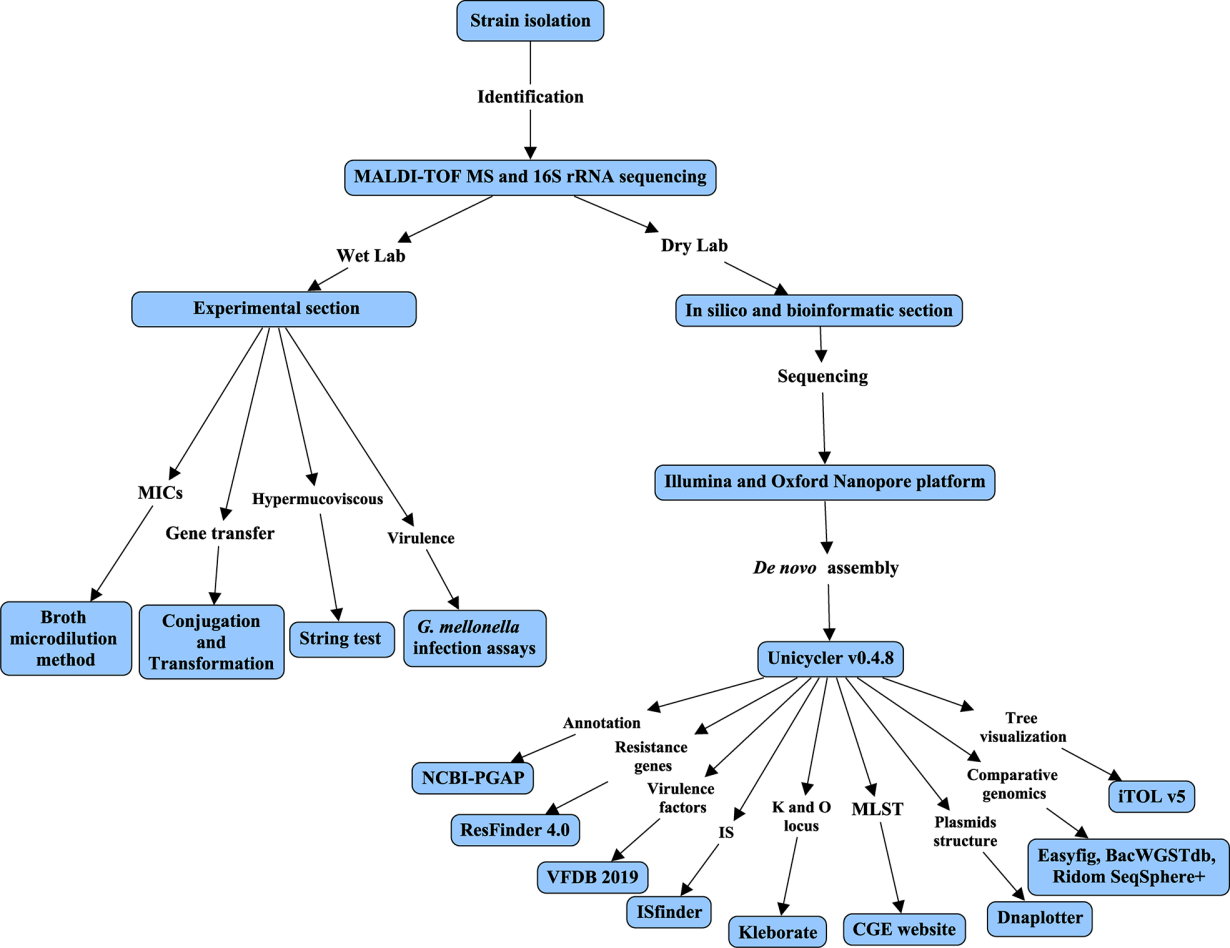
Flow chart of this study. The flow chart was constructed using CmapTools v6.04. The flow chart was divided into Wet and Dry labs based on experiments or bioinformatic analysis.

A flow chart (**Figure 1**) was constructed to detail all experiments and procedures associated with this study using CmapTools v6.04 (https://cmap.ihmc.us) (Behzadi and Gajdacs, 2021). Based on different contents of experiments and bioinformatic analysis, this study was divided into two parts, consisting of Wet Lab and Dry Lab.

### Bacterial isolation and identification

*K. pneumoniae* strain (KPTCM) was isolated from one patient with BSIs and liver abscess during the routine diagnostic in the hospital in Hangzhou, China in January 2022. No history of travel was reported. Unfortunately, liver abscess developed rapid and the outcome was death. According to the clinical symptoms of host, KPTCM may be a relatively hypervirulent strain and was chosen for further analysis. Isolate identification was performed by matrix-assisted laser desorption ionization-time of flight mass spectrometry ([MALDI-TOF MS] Bruker Daltonik GmbH, Bremen, Germany) and further confirmed by 16S rRNA gene-based PCR followed by sequencing (Huada, China).

### Antimicrobial susceptibility testing

Minimum inhibitory concentrations (MICs) against multiple antimicrobial agents were determined using broth microdilution method and interpreted according to Clinical and Laboratory Standards Institute (CLSI) 2021 guidelines. Antibiotics, including imipenem (IPM), meropenem (MEM), ertapenem (ETP), amikacin (AMI), ciprofloxacin (CIP), colistin (COL), tigecycline (TGC), ceftazidime/avibactam (CAZ-AVI), ceftazidime (CAZ), cefepime (FEP), meropenem-vaborbactam (M/V) and cefiderocol (CFD), were investigated in this study. *Escherichia coli* ATCC 25922 served as the quality control strain.

### Conjugation and chemical transformation experiments

To determine the transferable ability of plasmid carrying *bla*_OXA-232_, conjugation experiments using *E. coli* J53 (sodium azide resistant) as the recipient strain were carried out using film mating method (Yang et al., 2021b). Transconjugants were screened on Mueller–Hinton agar plates containing sodium azide (100 mg/L) and meropenem (2 mg/L). Donor or recipient bacteria alone was used as the control culture (Liu et al., 2021). The identity of putative transconjugants was confirmed *via* PCR and MALDI-TOF MS. Experiments were performed in triplicate independently.

Chemical transformation was further performed when conjugation failed. Plasmid harboring *bla*_OXA-232_ gene was transferred into *E. coli* DH5α *via* chemical transformation with imipenem (0.125 mg/L) for selection to yield *E. coli* DH5α:pOXA-232 (*E. coli* DH5α containing OXA-232 plasmid) (Froger and Hall, 2007). The MICs for carbapenems were measured and compared to the MICs values of *E. coli* DH5α.

### Hypermucoviscous phenotype determination

String test assay was performed based on previous description (Shen et al., 2020a). Briefly, KPTCM strain was cultured on a sheep blood agar plate at 37 °C for an overnight culture followed by streaking an inoculation loop through a colony in the next day. Formation of a viscous string > 5 mm in length was considered as a positive phenotype.

### *Galleria mellonella* infection model

*Galleria mellonella* infection model was used to assess virulence as previously described (Menard et al., 2021; Zhao et al., 2021). In brief, log-phase bacteria were centrifuged, resuspended using phosphate-buffered saline (PBS) and then diluted 10-fold to yield 1 × 10^8^ colony forming units/mL (CFU/mL). An aliquot of 10 µL (1 × 10^6^ CFU) was injected into *G. mellonella* larvae (n = 10, 0.2–0.3 g; Yuejiayin, Tianjin, China) using a Hamilton syringe (Hamilton, USA). *G. mellonella* larvae were incubated at 37°C and survival was recorded every 12 h and monitored for 72 h in total. *K. pneumoniae* NTUH-2044 was used as a positive control, and one cKP isolate (*K. pneumoniae* ATCC700603) served as a virulence negative control (Liao et al., 2020). Experiments were carried out in triplicate independently.

### Whole genome sequencing and bioinformatics analysis

Genomic DNA was extracted from KPTCM strain using a Qiagen minikit (Qiagen, Hilden, Germany) upon the manufacturer’s recommendations with minor modifications. Whole genome was sequenced using both Illumina HiSeq platform (Illumina, San Diego, CA, USA) and Oxford Nanopore MinION platform (Nanopore, Oxford, UK). *De novo* assembly of the reads of Illumina and MinION was constructed using Unicycler v0.4.8 (Wick et al., 2017). Genome sequence annotation was performed using National Center for Biotechnology Information (NCBI) Prokaryotic Genome Annotation Pipeline (PGAP) (http://www.ncbi.nlm.nih.gov/genome/annotation_prok/) (Tatusova et al., 2016). Antimicrobial resistance determinants were identified using ABRicate program (https://github.com/tseemann/abricate) based on ResFinder database (http://genomicepidemiology.org/) (Zankari et al., 2012). Bacterial virulence factors were identified *via* virulence factor database (VFDB, http://www.mgc.ac.cn/VFs/) (Liu et al., 2022). Insertion sequences (ISs) were identified using ISfinder (Siguier et al., 2006). *wzi*, K and O antigen loci were analyzed using Kleborate with *Kaptive* (Lam et al., 2021; Lam et al., 2022). Plasmid structure was visualized using DNAplotter (https://www.sanger.ac.uk/tool/dnaplotter/). Plasmid comparison with p47733_OXA_181 (Genbank accession number: CP050368) (Chudejova et al., 2021) was performed using Easyfig (Sullivan et al., 2011) and visualized with Adobe Illustrator. Phylogenetic analysis of 109 ST15 *K. pneumoniae* strains was performed using core genome multilocus sequence typing (cgMLST) on the Ridom SeqSphere+ server (Chen et al., 2021b). MLST, replicon type, single-nucleotide polymorphism (SNP) strategies, and further cgMLST analysis for 45 OXA-232-producing *K. pneumoniae* strains were conducted using BacWGSTdb server (Ruan et al., 2020; Feng et al., 2021). Generation tree file was visualized using the Interactive Tree of Life (iTOL, https://itol.embl.de/) (Letunic and Bork, 2021).

### Statistical analysis

*G. mellonella* survival rates were evaluated through Kaplan–Meier survival curves. Statistical significance was analyzed using a log-rank (Mantel–Cox) test with GraphPad Prism 8.0.2 software (GraphPad Software, San Diego California USA). *P* values of < 0.05 were considered statistically significant.

## Results

### MICs, antimicrobial resistance and virulence determinants

Antimicrobial susceptibility testing revealed KPTCM strain possessed a multidrug-resistant (MDR) profile with meropenem and ertapenem MICs of 4 mg/L and 8 mg/L, respectively. Furthermore, KPTCM strain exhibited resistance to ceftazidime (> 32 mg/L), cefepime (> 64 mg/L), ciprofloxacin (> 32 mg/L) and amikacin (> 128 mg/L). However, it was still susceptible to imipenem (1 mg/L), meropenem-vaborbactam (2 mg/L), cefiderocol (0.25 mg/L), ceftazidime/avibactam (0.5 mg/L), colistin (0.5 mg/L) and tigecycline (1 mg/L).

Analysis of the genome of KPTCM strain revealed that, in addition to co-harboring chromosomal *bla*_CTX-M-15_ and plasmid-mediated *bla*_OXA-232_, a series of genes conferring resistance to β-lactams (*bla*_SHV-106_, *bla*_SHV-12_ and blaTEM-1B), aminoglycosides (*strAB* and *rmtF*), sulfonamides (*sul2*), trimethoprim/sulfamethoxazole (*dfrA14*), quinolones (*qnrB1*), rifampicin (*arr-2*) and chloramphenicol (*catB*) were also identified (**Table 1**).

**Table 1:** Molecular characterization of genome from KPTCM strain.

Genome	Replicon	Size (bp)	GC content	Resistance genes	Accession numbers
chromosome	ND	5,186,161	57.36%	*bla*_CTX-15_, *bla*_SHV-106_, *oqxA6*, *oqxB20*, *fosA6*	*CP097385*
pKPTCM-1	IncFIB, IncHI1B	167,179	50.59%	ND	*CP097386*
pKPTCM-2	IncFIB	128,536	50.06%	*catB*, *arr-2*, *rmtF*	*CP097387*
pKPTCM-3	IncFII	116,556	51.44%	*dfrA14*, *qnrB1*	*CP097388*
pKPTCM-4	IncFII, IncX3	50,463	50.86%	*strAB*, *bla*_SHV-12_, *sul2*, *bla*_TEM-1B_	*CP097389*
pKPTCM-5	ColRNAI	9,730	53.22%	ND	*CP097390*
pKPTCM-6	ColKP3	6,141	52.19%	*bla*_OXA-232_	*CP097391*
pKPTCM-7	ND	5,640	49.96%	ND	*CP097392*
pKPTCM-8	Col440I	4,510	44.70%	ND	*CP097393*
pKPTCM-9	ColRNAI	3,770	41.74%	ND	*CP097394*

ND, Not detected.

Many virulence factors have been observed in KPTCM strain, including *rmpA2*, *iucABCD* and *iutA* gene cluster, which is responsible for encoding aerobactin. Furthermore, colibactin (*clbF* and *clbP*), yersiniabactin (*ybtAEPQSTUX*, *irp1*, *irp2* and *fyuA*), type 3 fimbriae (*mrkABCDFHIJ*) and genes encoding iron uptake function were further identified.

### Multilocus sequence typing, *wzi* type, KL and OCL

Based on the MLST scheme, KPTCM strain was typed into ST15. *Kaptive* showed Wzi, an outer membrane protein lectin, was classified as Wzi93. Furthermore, KPTCM strain contained O locus 1 (O1), matching the 98.51% nucleotide identity. The K locus in KPTCM strain was found to be KL112, to which it matches with an overall nucleotide identity of 98.65%.

### Transferability of plasmid harboring *bla*_OXA-232_

Mating assays were performed to investigate the transferability of *bla*_OXA-232_, however, *bla*_OXA-232_ failed to transfer to the recipient strain through conjugation. Concerning chemical transformation, MICs of *E. coli* DH5α:pOXA-232 to imipenem, meropenem and ertapenem rose to 1 mg/L, 0.25 mg/L and 2 mg/L, respectively, which was higher than that of *E. coli* DH5α strain (imipenem 0.06 mg/L, meropenem 0.03 mg/L and ertapenem 0.03 mg/L, respectively).

### Virulence assessment of KPTCM strain

**Figure 2:**
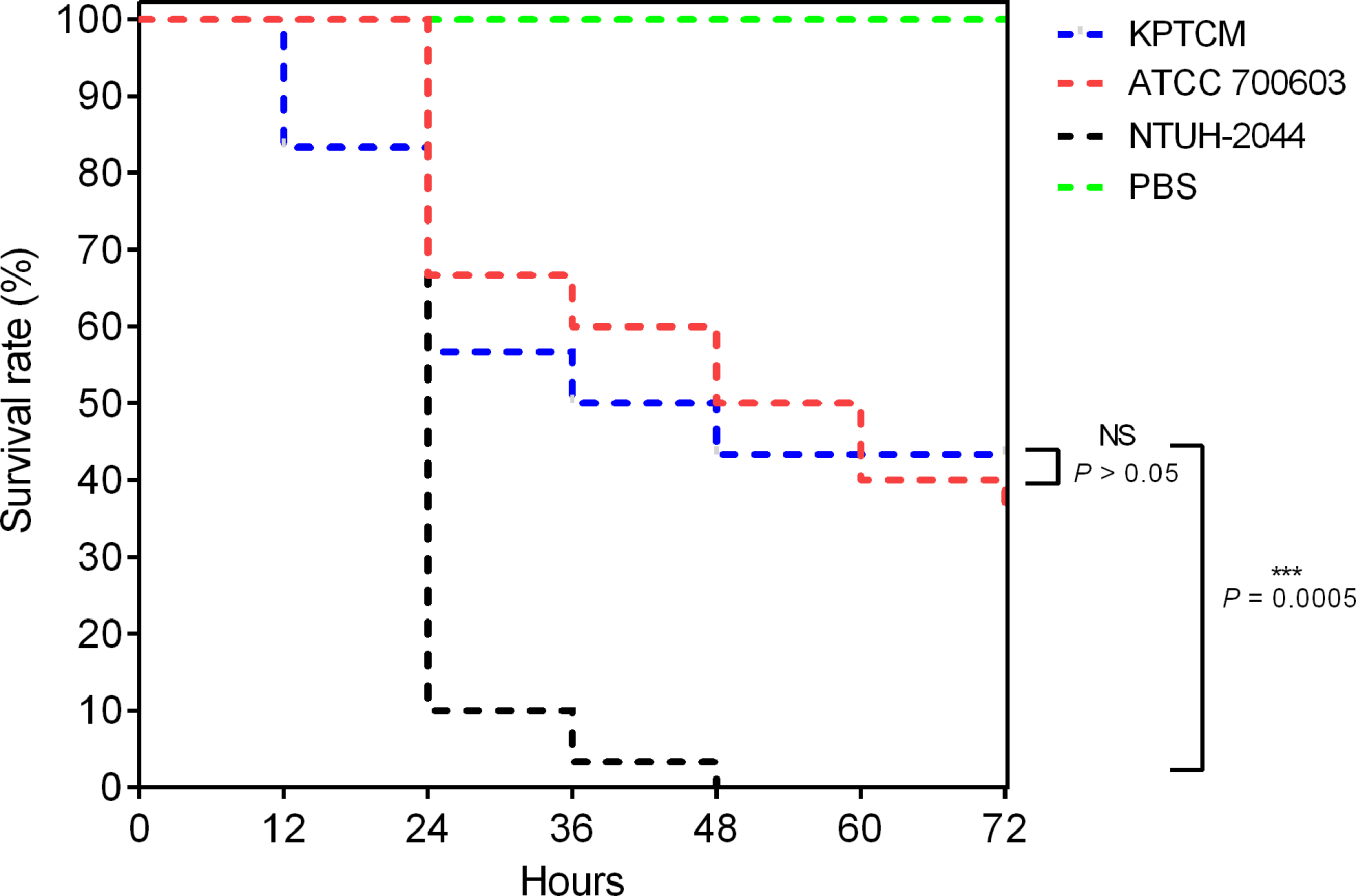
Survival curve in the *G. mellonella* infection model. *G. mellonella* larvae (n = 10) were inoculated with 10^6^ colony-forming units (CFU). Survival was recorded every 12 h for 72 h. *K. pneumoniae* NTUH-2044 was used as a positive control, and classic *K. pneumoniae* ATCC700603 served as a virulence negative control. Data from three independent experiments are shown. NS: No significance, *P* > 0.05; ***:*P* = 0.0005.

To explore the virulence level of KPTCM strain, virulence assays of string test and *G. mellonella* infection model were performed. As a result, negative phenotype was observed for string test experiment. In addition, *G. mellonella* infection model indicated virulence of KPTCM strain was significantly lower than standard hypervirulent isolate NTUH-2044 (*P* = 0.0005) but slightly higher than *K. pneumoniae* ATCC700603, used as negative control (**Figure 2**). However, no statistical significance was observed between KPTCM and *K. pneumoniae* ATCC700603 strains (*P* > 0.05).

### Genomic characterization of the chromosome

The hybrid assembly of both Illumina and MinION reads showed KPTCM strain contains a 5,186,161-bp circular chromosome with GC content of 57.36% (**Table 1**). The *bla*_CTX-M-15_ gene was embedded in the chromosome *via* IS*Ecp1*-based transposon Tn*2012*. Moreover, two amino acid mutations of *gyrA* (S83F) and *parC* (S80I) were identified, which confer resistance to fluoroquinolones.

### Plasmids in KPTCM strain

Nine plasmids were solved in KPTCM clinical strain, namely pKPTCM-1 to pKPTCM-9, with sizes from 3,770-bp to 167,179-bp and GC contents ranging from 41.74% to 53.22% (**Table 1**). pKPTCM-1 is a *rmpA2*-related pLVPK-like virulence plasmid with two replicons (IncFIB and IncHI1B). pKPTCM-2, pKPTCM-3 and pKPTCM-4 plasmids were found to be MDR plasmids harboring various resistance genes. pKPTCM-6 is one ColKP3-type plasmid with one carbapenem resistance gene, *bla*_OXA-232_. However, no resistance genes were identified in pKPTCM-5, pKPTCM-7, pKPTCM-8 and pKPTCM-9.

### Genetic features of pLVPK-like virulence plasmid pKPTCM-1

**Figure 3:**
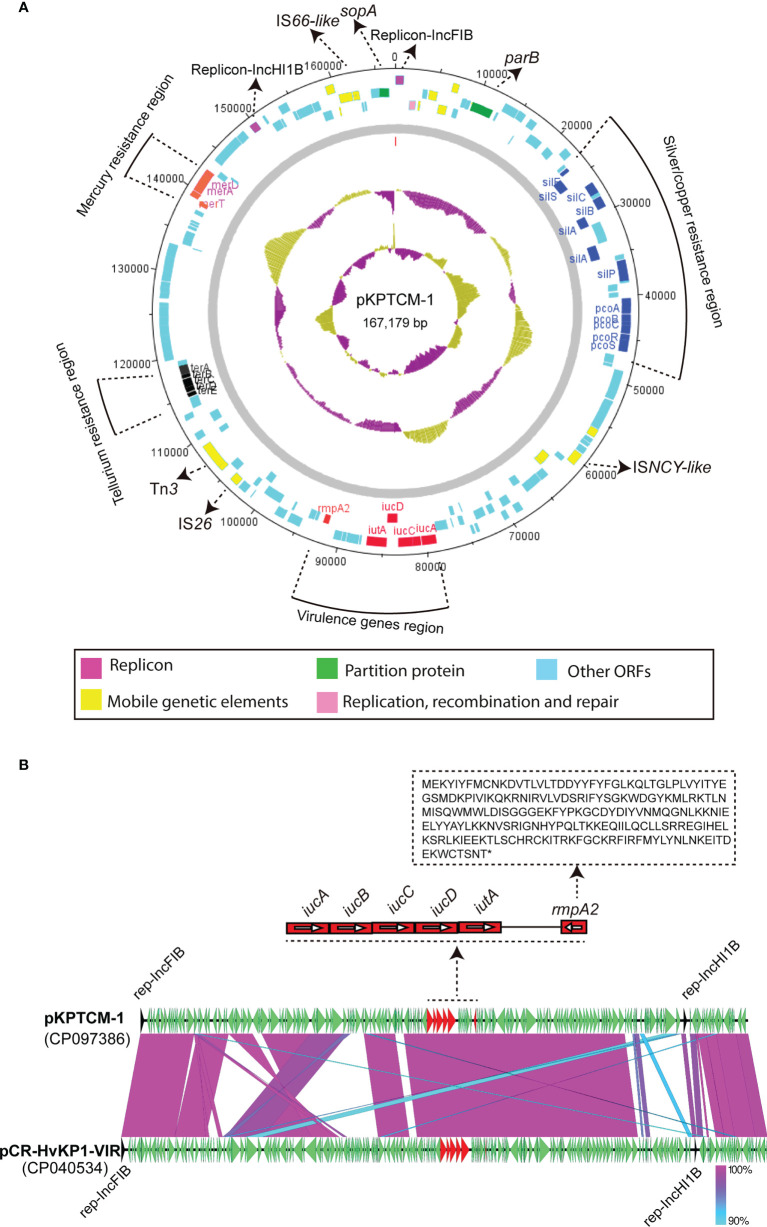
Circular map of pKPTCM-1 and comparison with pCR-HvKP1-VIR plasmid. **(A)** Circular map of pKPTCM-1 plasmid. Arrows show the direction of transcription of open reading frames (ORFs). Virulence genes region (red), tellurium resistance region (black), silver/copper resistance region (blue) and mercury resistance region (purple) are shown in different colors. ORFs of replicon, partition protein, mobile genetic elements (MGEs), replication, recombination and repair were further labeled. Light blue filled boxes represent other ORFs. **(B)** Structure of pKPTCM-1 compared with plasmid pCR-HvKP1-VIR (GenBank accession number: CP040534). The sequence of *rmpA2* with the frameshift mutation was shown. Shades indicate regions with 90% to 100% identity.

KPTCM clinical isolate harbored a 167,179-bp pLVPK-like virulence plasmid, namely pKPTCM-1, which was 99.65% identical to 177,790-bp pCR-HvKP1-VIR plasmid (GenBank accession number: CP040534) at 91% coverage (**Figure 3A**). pKPTCM-1 had an average GC content of 50.59% and consisted of four different regions, including virulence gene region, tellurium resistance region, silver/copper resistance region and mercury resistance region. *rmpA2* and *iucABCD*-*iutA* gene cluster were located in the virulence gene region. In addition, frameshift mutation was observed in *rmpA2* gene (**Figure 3B** and **Figure S1**).

### IS*26*-mediated plasmids fusion of pKPTCM-4

**Figure 4:**
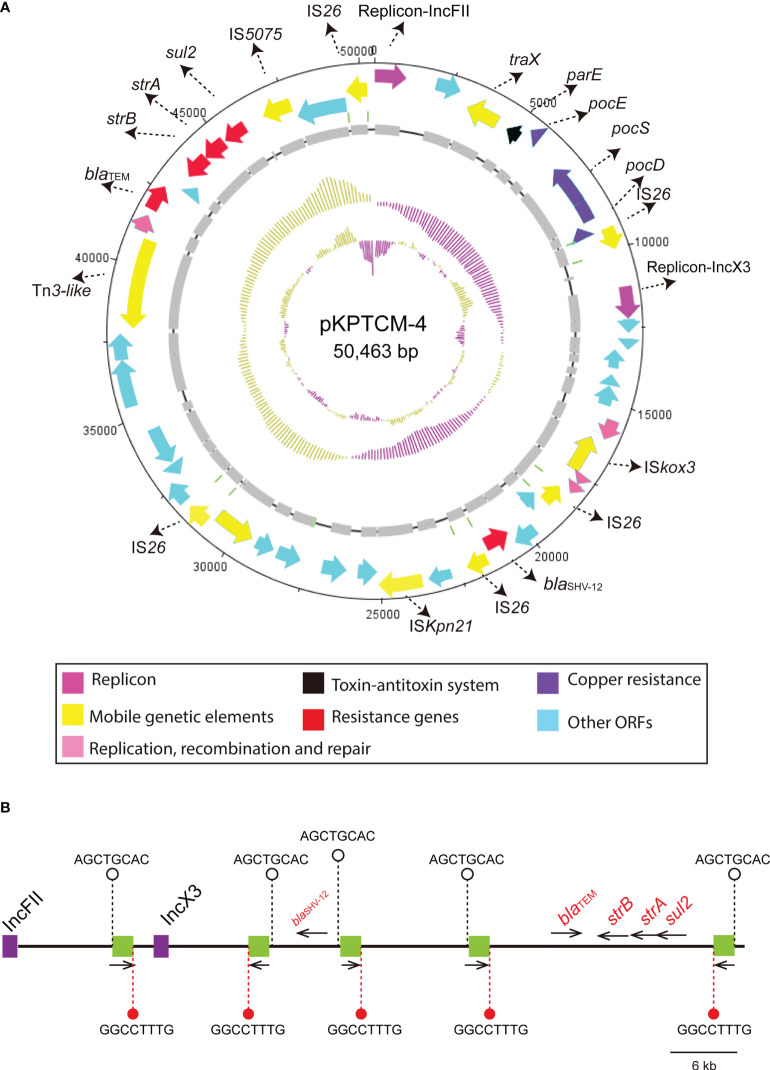
Circular map of pKPTCM-4 and IS*26* distribution. **(A)** Circular map of pKPTCM-4 plasmid. Arrows show the direction of transcription of ORFs. Red arrows indicate the antimicrobial resistance genes. Pink arrows indicate the replicons. ORFs of resistance genes, MGEs, copper resistance, recombination and repair were further labeled. **(B)** IS*26* elements are shown as green filled boxes. 8-bp target site duplications ([TSD] AGCTGCAC) were labeled on the left side, and the other 8-bp TSD (GGCCTTTG) were labeled on the right side of the IS*26*. Resistance genes were labeled as red with black thin arrows indicating ORF directions.

pKPTCM-4 is an IncFII-IncX3-typed plasmid with GC content of 50.86% (**Table 1**). Five resistance genes and five IS*26* genetic elements were identified in pKPTCM-4 plasmid (**Figure 4A**). In addition to IS*26*, IS*5075*, IS*Kpn21* and Tn*3*-like mobile genetic elements were also found. All resistance genes (*strAB*, *bla*_SHV-12_, *sul2* and *bla*_TEM-1B_) were flanked by various mobile genetic elements. Moreover, 8-bp target site duplications ([TSD] AGCTGCAC) were observed upstream of all five IS*26* genetic elements. The other 8-bp TSD (GGCCTTTG) were identified downstream of five IS*26* in pKPTCM-4 (**Figure 4B**).

### Genetic features of ColKP3-typed pKPTCM-6 plasmid

**Figure 5:**
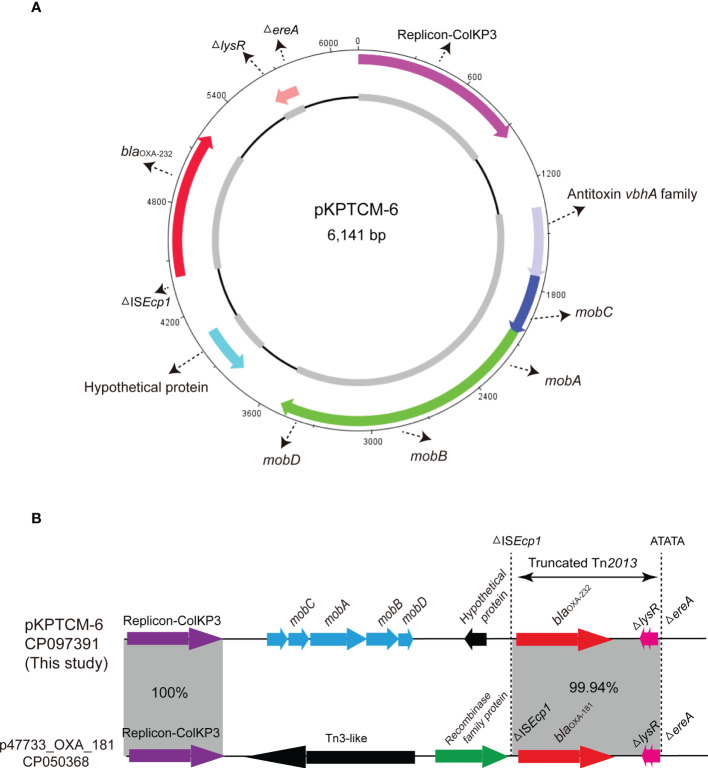
Circular map of pKPTCM-6. **(A)** Circular map of pKPTCM-6 plasmid. Arrows show the direction of ORFs. Red arrow indicate the *bla*_OXA-232_ gene. Pink arrow indicate the replicon. Light blue filled box represents hypothetical protein. Dark blue and green arrows indicate *mobC*, *mobA*, *mobB* and *mobD*. **(B)** Linear maps and comparison of the pKPTCM-6 and p47733_OXA_181 plasmids. Arrows show the direction of transcription of ORFs. Resistance genes are shown in red. The truncated Tn*2013* was labeled with 5-bp TSD (ATATA). Homologous segments (representing ≥99.9% sequence identity) are indicated by grey shading.

*bla*_OXA-232_ gene was located in a small 6,141-bp plasmid, designated pKPTCM-6 with ColKP3-typed replicon (**Table 1**). The plasmid was composed of *repA*, *mob* related genetic elements, ΔIS*Ecp1*, *bla*_OXA-232_, Δ*lysR* and Δ*ereA* (**Figure 5A**). A truncated Tn*2013* transposon structure was identified with 5-bp TSD (ATATA) on the right side (**Figure 5B**). Moreover, another ColKP3-typed plasmid p47733_OXA_181 (a *bla*_OXA-181_-carrying plasmid) showed 51% coverage and 99.9% identity to pKPTCM-6. The genetic environment between *bla*_OXA-232_ and *bla*_OXA-181_ was identical and both flanked by ΔIS*Ecp1* and Δ*lysR*-Δ*ereA* (**Figure 5B**).

### Comparative genomics analysis of 109 ST15 *K. pneumoniae* strains and 45 OXA-232-producing *K. pneumoniae* strains

**Figure 6:**
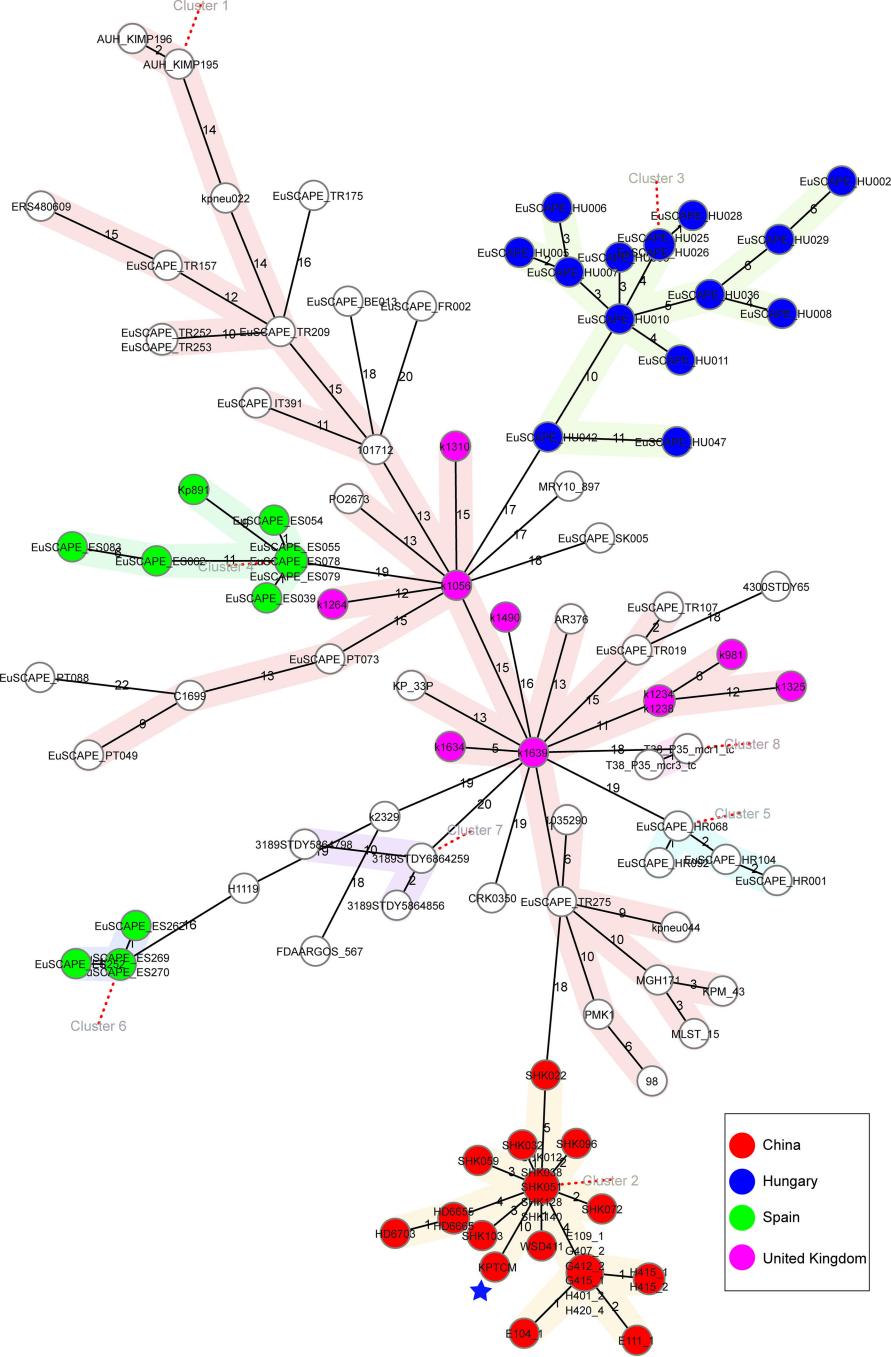
Phylogenetic analysis of 109 ST15 *K. pneumoniae* strains. cgMLST was conducted based on the 2358 alleles using Ridom SeqSphere+ server with cluster alert distance set at 15. Strains collected from China, Hungary, Spain and United Kingdom were shown as different colors. The blue pentagram indicated the position of KPTCM strain.

To analyse the characteristics of ST15 *K. pneumoniae* strains with close genetic relationship, genomes in public databases were searched and downloaded based on 200 SNPs threshold using BacWGSTdb server. To further study the characteristics of 109 ST15 *K. pneumoniae* strains from different countries, comparative genomics analysis was performed *via* Ridom SeqSphere+ server. All information of 109 ST15 *K. pneumoniae* strains is shown in **Table S1**. Based on the cgMLST tree, data showed ST15 *K. pneumoniae* isolates were mainly isolated from China (23.85%, 26/109), followed by Hungary (13.76%, 15/109), Spain (11.01%, 12/109) and United Kingdom (9.17%, 10/109) (**Figure 6**). ST15 *K. pneumoniae* strains collected from China and Hungary were shown in cluster 2 and 3, respectively. However, the number of strains isolated from other countries, such as Turkey, Nepal, Lebanon and Switzerland, was relatively small (**Table S1**).

**Figure 7:**
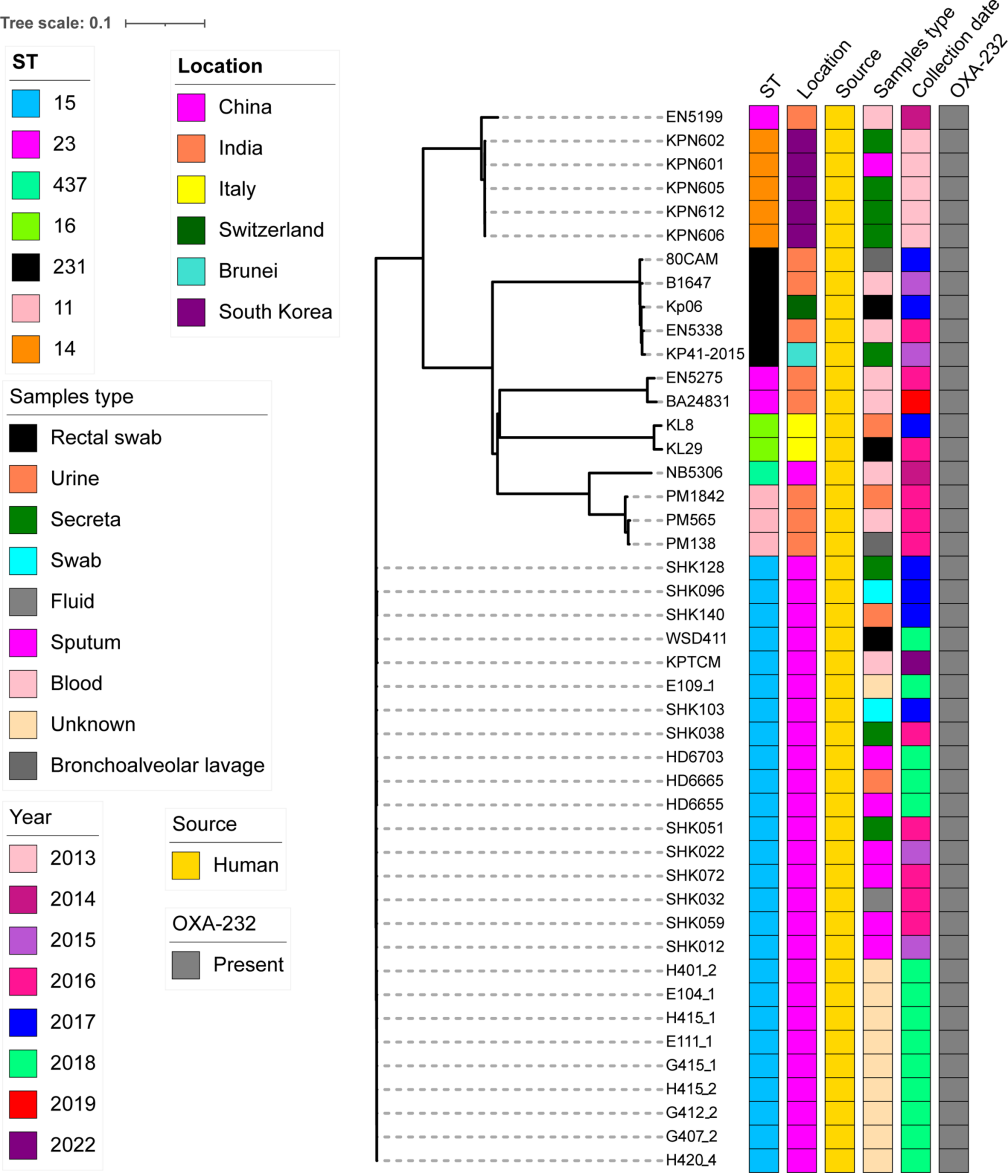
cgMLST analysis for 45 OXA-232-producing *K. pneumoniae* strains using BacWGSTdb server and visualized with iTOL v5. 26 OXA-232-producing ST15 *K. pneumoniae* strains from cluster 2 in Figure 6 and other 19 OXA-232-producing ST15 *K. pneumoniae* strains which were further searched from the published data. Isolates names, STs, locations, isolation date and collection sources are shown. The filled boxes reveal the various characteristics in different colors. The Genbank accession numbers of all 45 OXA-232-producing ST15 *K. pneumoniae* strains are shown in **Table S2**.

Other 19 OXA-232-producing *K. pneumoniae* strains were further searched based on published reports, the features of all 45 OXA-232-producing *K. pneumoniae* strains were analyzed using BacWGSTdb server. Interestingly, all *K. pneumoniae* strains carrying OXA-232 plasmid belonged to ST15 in China. However, STs in several countries, such as India (mainly ST11, ST23 and ST231), South Korea (ST14) and Italy (ST16), were quite different (**Figure 7**).

Of note, our data revealed that the first ST15 *K. pneumoniae* strain was isolated in 2015 in China. Strains were collected from several different sources, including blood, sputum, fluid, swab, urine, rectum and other sites (**Figure 7**). Moreover, SNP analysis was performed for the 26 OXA-232-producing ST15 strains from China. SNP strategy showed 28-222 SNPs differences among 26 ST15 *K. pneumoniae* isolates.

## Discussion

The presence of carbapenemase-producing *K. pneumoniae* has become dominant in several countries, and it is being increasingly considered a quite important nosocomial pathogen (Yang et al., 2021c). More importantly, BSIs caused by CRKP present critical problems in terms of clinical therapy (Xiao et al., 2020). Hypervirulent *K. pneumoniae* strains could also cause serious infections among healthy individuals (Sewunet et al., 2021). These hypervirulent *K. pneumoniae* strains are usually resistant to antimicrobials agents and become high-risk clones in the hospital (Sewunet et al., 2021).

Mobile genetic elements, including ISs, integrons, and transposons, play a particularly important role in the resistance gene transfer among different species (Gorbunova et al., 2021). One interesting finding from this study is that *bla*_CTX-M-15_ was located in the chromosome, which was mediated by IS*Ecp1*-based transposon Tn*2012*. IS*Ecp1* transposase gene has 14-bp inverted repeats (IRs) and is more inclined to insert into targets with the 5-bp AT-rich sites. IS*Ecp1* transposase gene is characterized by its capacity to transfer DNA fragments, such as resistance genes. Shu *et al*. reported pE109-1-CTX plasmid was found to harbor the resistance gene *bla*_CTX-M-15_ in China (Shu et al., 2019). Thus, it could be concluded that chromosomal *bla*_CTX-M-15_ in our strain may be derived from other plasmids mediated by IS*Ecp1*-based transposon Tn*2012.* Moreover, the genetic environment of *arr-2* gene cassette was found in a complex class 1 integron. Integrons are other mobile genetic elements, which could capture gene cassettes *via attl* and *attC*, further contributing to the global resistance crisis (Ghaly et al., 2021). More importantly, IS*26* plays a key role in the spread of resistance genes in Gram-negative bacteria (Harmer et al., 2014; Hua et al., 2020). A crucial feature of IS*26* is its capability to form co-integrate molecules that consists of distinct DNA segments (Hua et al., 2020). Based on the analysis of ISs and TSD, it was hypothesized that pKPTCM-4 with IncFII-IncX3 replicons was formed by five IS*26*-mediated co-integration events. Consequently, the joint role of diverse mobile genetic elements enables bacteria to undergo frequent genetic transposition and further leads to the MDR phenotype (Yang et al., 2021a).

Note that KPTCM was found to carry *rmpA2* and virulence-associated gene cluster (*iucABCD-iutA*), the virulence potential was further evaluated. Interestingly, hypermucoviscous phenotype was negative. Moreover, *rmpA* and *rmpA2* genes have been identified to be strongly associated with the hypermucoviscous phenotype. Nevertheless, *rmpA2* gene in KPTCM strain has indel mutations causing frameshifts, further rendering the gene product dysfunctional (Shen et al., 2020a). In the current study, the low virulence maybe is caused by frameshifts of *rmpA2*. Thus, screening for *rmpA* and *rmpA2* genes may need to be followed by sequencing to ensure their integrity, then it could be associated with hypervirulent phenotype. Interestingly, the patient developed a liver abscess caused by KPTCM strain and the outcome was death in a short time. Based on its associated clinical disease and outcome, the KPTCM strain should be considered as a relatively hypervirulent strain. However, a huge difference was observed when compared with many phenotypic experiments. Consequently, our results manifested the complexity and difficulty with the current knowledge for the definition of hypervirulence in *K. pneumoniae*.

The OCL and KL gene clusters, which are responsible for the biosynthesis of the outer core of lipooligosaccharide and capsule, are potentially useful epidemiological markers and offer a key role and potential target for vaccine development (Wyres et al., 2020). In the current study, KL112 had a quite high identity but with few reports. Furthermore, analysis of the population structure of the strains with various STs showed transmission among diverse countries. Based on previous reports, various STs have been reported in OXA-232-producing *K. pneumoniae* strains in many countries, including UK (mainly ST14, ST147, and ST231) (Findlay et al., 2017), Italy (ST16) (Avolio et al., 2017), Switzerland (ST231) (Mancini et al., 2018) and Tunisia (ST147) (Lahlaoui et al., 2017). However, our data revealed CRKP isolates harboring OXA-232-type carbapenemase plasmid usually belong to ST15 in China. The ST15 clones in China seem to be different from those in other areas of the world in view of the allelic differences and are mainly disseminated between Zhejiang and Shanghai (two geographically adjacent regions in China) but with slight evolution based on the SNP differences.

However, some limitations in this study remain, including the use of an insect larvae model might not be the most suitable for this type of virulence assessment. Thus, a murine infection model would be more suitable. Additionally, at present, we are not able to confirm whether resistance plasmids in KPTCM strain could be transferred to other recipient bacteria *via* conjugation. Finally, virulence-associated studies are required to find the direct or indirect connection between the carriage of the pLVPK-like virulence plasmid and hypervirulent phenotype in *K. pneumoniae*.

## Conclusion

This study provides a comprehensive description for the complete genome characteristics of a *wzi93*-KL112-O1 OXA-232-producing ST15 CRKP, which harbors a *rmpA2*-associated pLVPK-like virulence plasmid and chromosomal *bla*_CTX-M-15_ mediated by IS*Ecp1*-based transposon Tn*2012*. Considering that ST15 CRKP strains possess the strong plasmid carrying capacity (9 plasmids in KPTCM strain), they still have the potential to become hypervirulent *via* acquiring another type of virulence plasmid. Therefore, early detection of *K. pneumoniae* strains carrying chromosomal *bla*_CTX-M-15_, OXA-232 carbapenemase and pLVPK-like virulence plasmid is recommended to avoid the extensive spread of this high-risk clone in healthcare settings.

## Data availability statement

The datasets presented in this study can be found in online repositories. The names of the repository/repositories and accession number(s) can be found below: Genbank, PRJNA838426.

## Ethics statement

This study was approved by the local Ethics Committees of the Hospital with a waiver of informed consent due to this study mainly focused on bacterial genome and the retrospective nature of the study.

## Author contributions

CT and MX designed the experiments, analyzed the data, and wrote the initial manuscript. CT, MX, YB, and YZ performed the majority of the experiments. MX collected the bacteria. XF, LF, and SW supervised this study and reviewed and edited the paper. All authors contributed to the article and approved the submitted version.

## Funding

This work was supported by Medical Health Science and Technology Project of Zhejiang Provincial Health Commission (2022RC278), Natural Science Foundation of Zhejiang Province (LGF20H300003, LGF20H280002, LQ19H160002), Quzhou technology projects, China (2019K36).

## Acknowledgments

We thank Charlesworth Author Services for the help with revising the manuscript.

## Conflict of interest

The authors declare that the research was conducted in the absence of any commercial or financial relationships that could be construed as a potential conflict of interest.

## Publisher’s note

All claims expressed in this article are solely those of the authors and do not necessarily represent those of their affiliated organizations, or those of the publisher, the editors and the reviewers. Any product that may be evaluated in this article, or claim that may be made by its manufacturer, is not guaranteed or endorsed by the publisher.

## Supplementary material

Supplementary Figure 1 Sequence alignment of *rmpA2* genes. *rmpA2** indicates gene with frameshifts in pKPTCM-1 plasmid. *rmpA2* is the wild type in pK2044 of *K. pneumoniae* NTUH-2044.
