# Supplementary figures and images for: Whole genome sequencing of OXA-232-producing wzi93-KL112-O1 carbapenem-resistant Klebsiella pneumoniae in human bloodstream infection co-harboring chromosomal ISEcp1-based bla CTX-M-15 and one rmpA2-associated virulence plasmid
Source: Front Cell Infect Microbiol. 2022 Sep 29;12:984479. doi: 10.3389/fcimb.2022.984479 (PMC9560801; doi:10.3389/fcimb.2022.984479)

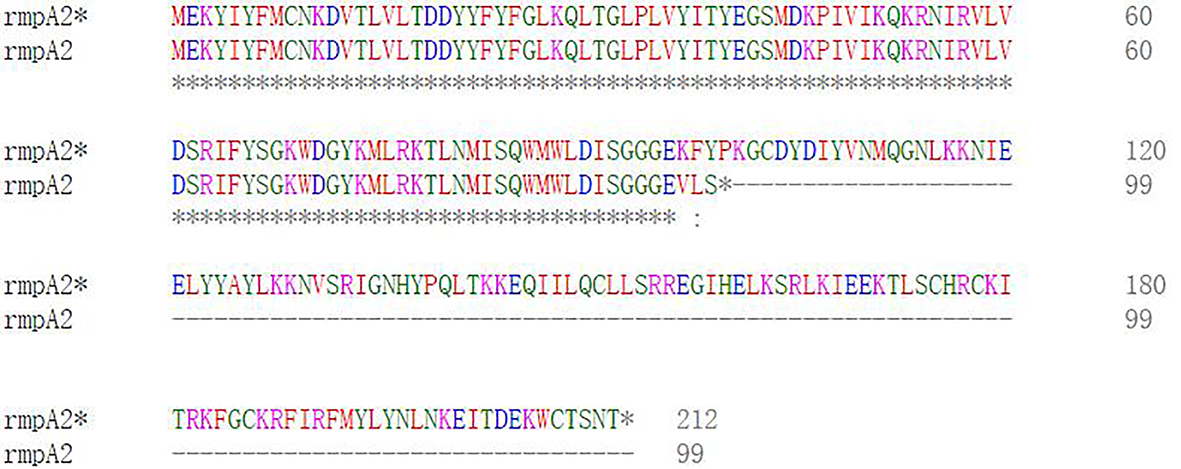

Supplement: Supplementary Figure 1 — Sequence alignment of rmpA2 genes. rmpA2* indicates gene with frameshifts in pKPTCM-1 plasmid. rmpA2 is the wild type in pK2044 of K. pneumoniae NTUH-2044. [file Image_1.tif]
