# Supplementary material for: Whole genome sequencing of OXA-232-producing wzi93-KL112-O1 carbapenem-resistant Klebsiella pneumoniae in human bloodstream infection co-harboring chromosomal ISEcp1-based bla CTX-M-15 and one rmpA2-associated virulence plasmid
Source: Front Cell Infect Microbiol. 2022 Sep 29;12:984479. doi: 10.3389/fcimb.2022.984479 (PMC9560801; doi:10.3389/fcimb.2022.984479)
Supplement: Supplementary file 2 [file Table_1.docx]

**Table S1. Close isolates information used in this study.**

| **Isolate** | **Accession**  **number** | **ST** | **Host** | **Isolation**  **source** | **Location** | **Collection**  **date** | **Resistance genes** | **Different alleles** |
| --- | --- | --- | --- | --- | --- | --- | --- | --- |
| SHK051 | SIZO01 | 15 | Human | secreta | China: Shanghai | 27-Feb-16 | ARR-2,aac(6')-Ib-Hangzhou,aph(3'')-Ib,aph(6)-Id,blaCTX-M-15,blaOXA-232,blaSHV-106,blaTEM-1B,dfrA14,fosA,oqxA,oqxB,qnrB1,rmtF,sul2 | 76 |
| SHK128 | SIZT01 | 15 | Human | secreta | China: Shanghai | 14-Jun-17 | ARR-2,aac(6')-Ib-Hangzhou,aph(3'')-Ib,aph(6)-Id,blaCTX-M-15,blaOXA-232,blaSHV-106,blaTEM-1B,dfrA14,fosA,oqxA,oqxB,qnrB1,rmtF,sul2 | 86 |
| SHK140 | SIZU01 | 15 | Human | urine | China: Shanghai | 29-Sep-17 | ARR-2,aac(6')-Ib-Hangzhou,aph(3'')-Ib,aph(6)-Id,blaCTX-M-15,blaOXA-232,blaSHV-106,blaTEM-1B,dfrA14,fosA,oqxA,oqxB,qnrB1,rmtF,sul2 | 92 |
| SHK022 | SIZL01 | 15 | Human | sputum | China: Shanghai | 2015/12/20 | ARR-2,aac(6')-Ib-Hangzhou,aph(3'')-Ib,aph(6)-Id,blaCTX-M-15,blaOXA-232,blaSHV-106,blaTEM-1B,dfrA14,fosA,oqxA,oqxB,qnrB1,rmtF,sul2 | 103 |
| SHK032 | SIZM01 | 15 | Human | Drainage of fluid | China: Shanghai | 2016/1/5 | ARR-2,aac(6')-Ib-Hangzhou,aph(3'')-Ib,aph(6)-Id,blaCTX-M-15,blaOXA-232,blaSHV-106,blaTEM-1B,dfrA14,fosA,oqxA,oqxB,qnrB1,rmtF,sul2 | 105 |
| SHK038 | SIZN01 | 15 | Human | secreta | China: Shanghai | 2016/10/4 | ARR-2,aac(6')-Ib-Hangzhou,aph(3'')-Ib,aph(6)-Id,blaCTX-M-15,blaOXA-232,blaSHV-106,blaTEM-1B,dfrA14,fosA,oqxA,oqxB,qnrB1,rmtF,sul2 | 107 |
| WSD411 | CP045674 | 15 | Human | rectum | China | 26-Aug-18 | blaCTX-M-15,blaSHV-106,oqxA,oqxB | 107 |
| SHK059 | SIZP01 | 15 | Human | sputum | China: Shanghai | 2016/5/6 | ARR-2,aac(6')-Ib-Hangzhou,aph(3'')-Ib,aph(6)-Id,blaCTX-M-15,blaOXA-232,blaSHV-106,blaTEM-1B,dfrA14,fosA,oqxA,oqxB,qnrB1,rmtF,sul2 | 111 |
| SHK072 | SIZQ01 | 15 | Human | sputum | China: Shanghai | 26-Jul-16 | ARR-2,aac(6')-Ib-Hangzhou,aph(3'')-Ib,aph(6)-Id,blaCTX-M-15,blaOXA-232,blaSHV-106,blaTEM-1B,dfrA14,fosA,oqxA,oqxB,qnrB1,rmtF,sul2 | 111 |
| SHK096 | SIZR01 | 15 | Human | swab | China: Shanghai | 2017/10/23 | ARR-2,aac(6')-Ib-Hangzhou,aph(3'')-Ib,aph(6)-Id,blaCTX-M-15,blaOXA-232,blaSHV-106,blaTEM-1B,dfrA14,fosA,oqxA,oqxB,qnrB1,rmtF,sul2 | 112 |
| G412_2 | QRGJ01 | 15 | Human | - | China | 2018 | ARR-2,aac(6')-Ib-Hangzhou,aph(3'')-Ib,aph(6)-Id,blaCTX-M-15,blaOXA-232,blaSHV-106,blaTEM-1B,dfrA14,fosA,oqxA,oqxB,qnrB1,rmtF,sul2 | 113 |
| G415_1 | QRGI01 | 15 | Human | - | China | 2018 | ARR-2,aac(6')-Ib-Hangzhou,aph(3'')-Ib,aph(6)-Id,blaCTX-M-15,blaOXA-232,blaSHV-106,blaTEM-1B,dfrA14,fosA,oqxA,oqxB,qnrB1,rmtF,sul2 | 113 |
| H401_2 | QRGH01 | 15 | Human | - | China | 2018 | ARR-2,aac(6')-Ib-Hangzhou,aph(3'')-Ib,aph(6)-Id,blaCTX-M-15,blaOXA-232,blaSHV-106,blaTEM-1B,dfrA14,fosA,oqxA,oqxB,qnrB1,rmtF,sul2 | 113 |
| E104_1 | QRGM01 | 15 | Human | - | China | 2018 | ARR-2,aac(6')-Ib-Hangzhou,aph(3'')-Ib,aph(6)-Id,blaCTX-M-15,blaOXA-232,blaSHV-106,blaTEM-1B,dfrA14,fosA,oqxA,oqxB,qnrB1,rmtF,sul2 | 114 |
| G407_2 | QRGK01 | 15 | Human | - | China | 2018 | ARR-2,aac(6')-Ib-Hangzhou,aph(3'')-Ib,aph(6)-Id,blaCTX-M-15,blaOXA-232,blaSHV-106,blaTEM-1B,dfrA14,fosA,oqxA,oqxB,qnrB1,rmtF,sul2 | 114 |
| H420_4 | QRGE01 | 15 | Human | - | China | 2018 | ARR-2,aac(6')-Ib-Hangzhou,aph(3'')-Ib,aph(6)-Id,blaCTX-M-15,blaOXA-232,blaSHV-106,blaTEM-1B,dfrA14,fosA,oqxA,oqxB,qnrB1,rmtF,sul2 | 114 |
| SHK012 | SIZK01 | 15 | Human | sputum | China: Shanghai | 2015/10/25 | ARR-2,aac(6')-Ib-Hangzhou,aph(3'')-Ib,aph(6)-Id,blaCTX-M-15,blaOXA-232,blaSHV-106,blaTEM-1B,dfrA14,fosA,oqxA,oqxB,qnrB1,rmtF,sul2 | 114 |
| E109_1 | QRGN01 | 15 | Human | - | China | 2018 | ARR-2,aac(6')-Ib-Hangzhou,aph(6)-Id,blaCTX-M-15,blaOXA-232,blaSHV-106,blaTEM-1B,oqxA,oqxB,rmtF | 115 |
| E111_1 | QRGL01 | 15 | Human | - | China | 2018 | ARR-2,aac(6')-Ib-Hangzhou,aph(3'')-Ib,aph(6)-Id,blaCTX-M-15,blaOXA-232,blaSHV-106,blaTEM-1B,dfrA14,fosA,oqxA,oqxB,qnrB1,rmtF,sul2 | 115 |
| H415_1 | QRGG01 | 15 | Human | - | China | 2018 | ARR-2,aac(6')-Ib-Hangzhou,aph(3'')-Ib,aph(6)-Id,blaCTX-M-15,blaOXA-232,blaSHV-106,blaTEM-1B,dfrA14,fosA,oqxA,oqxB,qnrB1,rmtF,sul2 | 115 |
| H415_2 | QRGF01 | 15 | Human | - | China | 2018 | ARR-2,aac(6')-Ib-Hangzhou,aph(3'')-Ib,aph(6)-Id,blaCTX-M-15,blaOXA-232,blaSHV-106,blaTEM-1B,dfrA14,fosA,oqxA,oqxB,qnrB1,rmtF,sul2 | 115 |
| SHK103 | SIZS01 | 15 | Human | swab | China: Shanghai | 2017/10/27 | ARR-2,aac(6')-Ib-Hangzhou,aph(3'')-Ib,aph(6)-Id,blaCTX-M-15,blaOXA-232,blaSHV-106,blaTEM-1B,dfrA14,fosA,oqxA,oqxB,qnrB1,rmtF,sul2 | 116 |
| EuSCAPE_TR275 | UJYC01 | 15 | Human | Lower Respiratory Tract Secrection | Turkey | 2013 | aac(6')-Ib3,blaCMY-6,blaCTX-M-15,blaNDM-1,blaOXA-48,blaSHV-106,fosA,oqxA,oqxB,rmtC,sul1 | 149 |
| PMK1 | CP008929 | 15 | Human | Blood | Nepal | 16-Aug-11 | blaCTX-M-15,blaSHV-106,oqxA,oqxB | 153 |
| 98 | CDQG01 | 15 | Human | Tracheal aspiration | Nepal | 2012 | aac(6')-Ib-cr,aadA2,aph(3'')-Ib,aph(3')-Ia,aph(6)-Id,armA,blaCTX-M-15,blaNDM-1,blaOXA-1,blaSHV-106,blaTEM-1B,catA1,dfrA12,dfrA14,erm(B),fosA,mph(A),mph(E),msr(E),oqxA,oqxB,qnrB1,sul1,tet(D) | 154 |
| kpneu044 | UWYA01 | 15 | Human | hospital | Switzerland | 2012/4/7 | ARR-2,aac(3)-IIa,aac(6')-Ib-cr,aadA1,blaCTX-M-15,blaNDM-1,blaOXA-1,blaSHV-106,blaTEM-1B,dfrA14,ere(A),oqxA,oqxB,qnrB1,rmtC,sul1 | 154 |
| KPM_43 | JAAJSJ01 | 15 | Human | - | Lebanon | 2017/10/2 | aac(6')-Ib,aac(6')-Ib-cr,aph(3'')-Ib,aph(6)-Id,blaCMY-6,blaCTX-M-15,blaNDM-1,blaOXA-1,blaSHV-106,blaTEM-1B,fosA,oqxA,oqxB,qnrB9,rmtC,sul1,sul2 | 158 |
| MLST_15 | CP022127 | 15 | Human | wound | USA: Nevada | 2016/8/19 | blaCTX-M-15,blaSHV-106,oqxA,oqxB | 163 |
| AR376 | CP029137 | 15 | - | - | - | - | aac(3)-IIa,blaCTX-M-15,blaOXA-1,blaSHV-106,oqxA,oqxB | 169 |
| k1056 | FLIR01 | 15 | Human | Blood | United Kingdom | 2005 | blaCTX-M-15,blaOXA-1,blaSHV-106,blaTEM-1B,dfrA14,fosA,oqxA,oqxB,tet(A) | 171 |
| k1310 | FLHL01 | 15 | Human | Blood | United Kingdom | 2006 | aac(3)-IIa,aac(6')-Ib-cr,aph(6)-Id,blaCTX-M-15,blaOXA-1,blaSHV-106,blaTEM-1B,dfrA14,fosA | 171 |
| k1634 | FLAZ01 | 15 | Human | Blood | United Kingdom | 2007 | aac(3)-IIa,aac(6')-Ib-cr,blaCTX-M-15,blaOXA-1,blaSHV-106,fosA,oqxA,oqxB,tet(A) | 173 |
| k1639 | FLCA01 | 15 | Human | Blood | United Kingdom | 2007 | aac(3)-IIa,aac(6')-Ib-cr,blaCTX-M-15,blaOXA-1,fosA,oqxA,oqxB,qnrB1,tet(A) | 173 |
| MGH171 | NGTL01 | 15 | Human | - | USA: Massachusetts, Boston | 2015 | aac(6')-Ib3,blaCMY-44,blaCTX-M-15,blaSHV-106,fosA,oqxA,oqxB,rmtC,sul1 | 173 |
| EuSCAPE_IT391 | UKWS01 | 15 | Human | Blood | Italy | 2013 | aac(3)-IIa,aac(6')-Ib-cr,aph(6)-Id,blaCTX-M-15,blaOXA-1,blaSHV-106,blaTEM-1B,dfrA14,fosA,oqxA,oqxB,tet(A) | 175 |
| 4300STDY6542352 | UFCX01 | 15 | Human | - | Thailand | 2016 | aadA2,aph(3')-Ia,blaCTX-M-15,blaOXA-1,blaSHV-106,blaTEM-1B,dfrA12,fosA,mph(A),oqxA,oqxB,sul1,tet(A) | 176 |
| EuSCAPE_TR019 | UJSO01 | 15 | Human | Urine | Turkey | 2014 | aac(6')-Ib-cr,aph(3')-Ia,blaCTX-M-15,blaOXA-1,blaSHV-106,blaTEM-1B,fosA,mph(A),oqxA,oqxB,sul1,tet(A) | 176 |
| k1238 | FLIU01 | 15 | Human | Blood | United Kingdom | 2005 | aac(3)-IIa,aac(6')-Ib-cr,aph(6)-Id,blaCTX-M-15,blaOXA-1,blaSHV-106,blaTEM-1B,fosA,oqxA,oqxB | 177 |
| EuSCAPE_HR092 | UJHZ01 | 15 | Human | Urine | Croatia | 2014 | aac(3)-IId,aac(6')-Ib-cr,aadA1,aadA2,aph(3'')-Ib,aph(6)-Id,blaCTX-M-15,blaOXA-1,blaSHV-106,blaTEM-1B,blaVIM-1,dfrA12,dfrA14,fosA,mph(A),oqxA,oqxB,sul1,sul2 | 178 |
| EuSCAPE_TR107 | UJRB01 | 15 | Human | Urine | Turkey | 2014 | aac(6')-Ib-cr,aph(3')-Ia,blaCTX-M-15,blaOXA-1,blaSHV-106,blaTEM-1B,fosA,mph(A),oqxA,oqxB,sul1,tet(A) | 178 |
| EuSCAPE_HR068 | UJML01 | 15 | Human | Lower Respiratory Tract Secrection | Croatia | 2013 | aac(3)-IId,aac(6')-Ib,aadA1,aadA2,aph(3'')-Ib,aph(6)-Id,blaCTX-M-15,blaOXA-1,blaSHV-106,blaTEM-1B,blaVIM-1,dfrA12,dfrA14,fosA,mph(A),oqxA,oqxB,sul1,sul2 | 179 |
| EuSCAPE_ES269 | UISH01 | 15 | Human | Urine | Spain | 2014 | aac(6')-Ib-cr,aph(3')-Ia,blaCTX-M-15,blaOXA-1,blaOXA-48,blaSHV-106,blaTEM-1B,fosA,mph(A),oqxA,oqxB,sul1 | 180 |
| EuSCAPE_ES252 | ULEJ01 | 15 | Human | Other (abscess) | Spain | 2014 | aac(6')-Ib-cr,aph(3')-Ia,blaCTX-M-15,blaOXA-1,blaOXA-48,blaSHV-106,blaTEM-1B,fosA,mph(A),oqxA,oqxB,sul1 | 181 |
| EuSCAPE_ES262 | ULEW01 | 15 | Human | Other (abscess) | Spain | 2014 | aac(6')-Ib-cr,aph(3')-Ia,blaCTX-M-15,blaOXA-1,blaOXA-48,blaSHV-106,blaTEM-1B,fosA,mph(A),oqxA,oqxB,sul1 | 181 |
| EuSCAPE_HU029 | ULBV01 | 15 | Human | Wound Secretion | Hungary | 2014 | aac(3)-IIa,aac(6')-Ib3,aph(3'')-Ib,aph(6)-Id,blaCTX-M-15,blaOXA-1,blaSHV-106,blaTEM-1B,blaVIM-4,dfrA14,fosA,mph(E),msr(E),oqxA,oqxB,sul1,sul2 | 181 |
| k1234 | FLIV01 | 15 | Human | Blood | United Kingdom | 2005 | aac(6')-Ib-cr,blaOXA-1,blaSHV-106,fosA,oqxA,oqxB | 181 |
| 101712 | JSZJ01 | 15 | Human | wound | USA: Fort Sam Houston | 2007/10/7 | aac(3)-IIa,aac(6')-Ib-cr,aph(6)-Id,blaCTX-M-15,blaOXA-1,blaSHV-106,blaTEM-1B,dfrA14,fosA,oqxA,oqxB | 182 |
| 3189STDY6864259 | UFAA01 | 15 | Human | - | Pakistan | 2014 | aac(6')-Ib3,aadA1,aph(3'')-Ib,aph(6)-Id,armA,blaCTX-M-15,blaNDM-1,blaOXA-10,blaSHV-106,blaTEM-1B,blaVEB-5,dfrA1,fosA,oqxA,oqxB,sul1,sul2,tet(D) | 182 |
| EuSCAPE_HR001 | UJKO01 | 15 | - | - | - | - | aac(6')-Ib-cr,aadA1,aadA2,aph(3'')-Ib,aph(6)-Id,blaCTX-M-15,blaOXA-1,blaSHV-106,blaTEM-1B,blaVIM-1,dfrA12,dfrA14,fosA,mph(A),oqxA,oqxB,sul1,sul2 | 182 |
| k1325 | FLBR01 | 15 | Human | Blood | United Kingdom | 2006 | aac(3)-IIa,aac(6')-Ib-cr,aph(6)-Id,blaCTX-M-15,blaOXA-1,blaSHV-106,blaTEM-1B,fosA,oqxA,oqxB | 182 |
| k981 | FLEU01 | 15 | Human | Blood | United Kingdom | 2004 | aac(3)-IIa,aac(6')-Ib-cr,aph(6)-Id,blaCTX-M-15,blaOXA-1,blaSHV-106,blaTEM-1B,dfrA14,fosA,oqxA,oqxB | 182 |
| EuSCAPE_HU002 | ULCP01 | 15 | Human | Urine | Hungary | 2013 | aac(6')-Ib-cr,aph(3'')-Ib,aph(6)-Id,blaCTX-M-15,blaOXA-1,blaSHV-106,blaTEM-1B,blaVIM-4,dfrA14,fosA,mph(E),msr(E),oqxA,oqxB,sul1,sul2 | 183 |
| EuSCAPE_ES270 | ULCV01 | 15 | Human | Urine | Spain | 2013 | aac(3)-Ia,aac(6')-Ib-cr,aadA1,aadA2,aph(3')-Ia,blaCTX-M-15,blaOXA-1,blaOXA-48,blaSHV-106,blaTEM-1B,dfrA12,fosA,mph(A),oqxA,oqxB,sul1,sul2 | 185 |
| EuSCAPE_HR104 | UJIF01 | 15 | - | - | - | - | aac(3)-IId,aac(6')-Ib,aadA1,aadA2,aph(3'')-Ib,aph(6)-Id,blaCTX-M-15,blaOXA-1,blaSHV-106,blaTEM-1B,blaVIM-1,dfrA12,dfrA14,fosA,mph(A),oqxA,oqxB,sul1,sul2 | 185 |
| EuSCAPE_HU047 | ULCI01 | 15 | Human | Puncture Fluids | Hungary | 2013 | aac(3)-IIa,aac(6')-Ib-cr,aph(3'')-Ib,aph(6)-Id,blaCTX-M-15,blaOXA-534,blaSHV-106,blaTEM-1B,dfrA14,fosA,oqxA,oqxB,sul2 | 186 |
| EuSCAPE_TR209 | UJWU01 | 15 | Human | Wound Secretion | Turkey | 2013 | aac(3)-IIa,aac(6')-Ib3,aph(3'')-Ib,aph(6)-Id,blaCTX-M-15,blaIMP-1,blaOXA-1,blaSHV-106,blaTEM-1B,cmlA1,dfrA14,fosA,oqxA,oqxB,sul1,sul2,tet(A) | 187 |
| T38_P35_mcr1_tc | VIAK01 | 15 | Human | feces | Thailand | 2018 | aadA2,aph(3'')-Ib,aph(6)-Id,blaCTX-M-15,blaKPC-2,mcr-1.1,blaSHV-106,blaTEM-1B,catA1,dfrA12,fosA,mph(A),oqxA,oqxB,qnrB1,sul1,sul2 | 187 |
| T38_P35_mcr3_tc | VIAJ01 | 15 | Human | feces | Thailand | 2018 | aac(6')-Ib-cr,aadA2,aph(3'')-Ib,aph(6)-Id,blaCMY-2,blaCTX-M-15,blaKPC-2,blaOXA-1,blaSHV-106,catA1,dfrA12,dfrA14,fosA,mcr-3.1,mph(A),oqxA,oqxB,qnrB1,sul1,sul2,tet(A) | 188 |
| EuSCAPE_TR157 | UJRU01 | 15 | Human | Blood | Turkey | 2013 | aac(3)-IIa,aac(6')-Ib-cr,aph(3'')-Ib,aph(6)-Id,blaCTX-M-15,blaOXA-1,blaSHV-106,blaTEM-1B,dfrA14,fosA,oqxA,oqxB,sul2,tet(A) | 189 |
| KP_33P | JXJG01 | 15 | Human | Blood | Netherlands: Groningen | 12-Aug-10 | aac(3)-IIa,aac(6')-Ib-cr,aph(3'')-Ib,aph(6)-Id,blaCTX-M-15,blaOXA-1,blaSHV-106,blaTEM-1B,dfrA14,fosA,oqxA,oqxB,sul2,tet(D) | 189 |
| kpneu022 | UWWN01 | 15 | Human | hospital | Switzerland | 2010 | blaCTX-M-15,blaOXA-1,blaSHV-106,oqxA,oqxB,tet(A) | 189 |
| EuSCAPE_PT088 | UIXB01 | 15 | Human | Urine | Portugal | 2013 | aph(6)-Id,blaCTX-M-15,blaSHV-106,blaTEM-1B,fosA,oqxA,oqxB | 190 |
| EuSCAPE_SK005 | UKDU01 | 15 | Human | Urine | Slovakia | 2014 | aac(3)-IIa,aac(6')-Ib-cr,aph(3'')-Ib,aph(6)-Id,blaCTX-M-15,blaOXA-1,blaSHV-106,blaTEM-1B,dfrA14,fosA,oqxA,oqxB,sul2 | 190 |
| EuSCAPE_TR252 | UJXW01 | 15 | Human | Blood | Turkey | 2013 | aac(3)-IIa,aac(6')-Ib3,aph(3'')-Ib,aph(6)-Id,blaCTX-M-15,blaIMP-1,blaOXA-1,blaSHV-106,blaTEM-1A,cmlA1,dfrA14,dfrA22,fosA,oqxA,oqxB,rmtD,sul1,sul2,tet(A) | 190 |
| EuSCAPE_TR253 | UJXN01 | 15 | Human | Blood | Turkey | 2013 | aac(3)-IIa,aac(6')-Ib3,aph(3'')-Ib,aph(6)-Id,blaCTX-M-15,blaIMP-1,blaOXA-1,blaSHV-106,blaTEM-1A,cmlA1,dfrA14,dfrA22,fosA,oqxA,oqxB,rmtD,sul1,sul2,tet(A) | 190 |
| 1035290 | RDWC01 | 15 | - | - | Turkey | 2017 | aac(6')-Ib3,blaCMY-6,blaCTX-M-15,blaNDM-1,blaSHV-106,fosA,oqxA,oqxB,rmtC,sul1 | 191 |
| CRK0350 | PTFL02 | 15 | Homo sapiens | urine | USA: Ohio | 2012 | aac(6')-Ib-cr,blaCTX-M-15,blaKPC-2,blaOXA-1,blaOXA-9,blaSHV-106,blaTEM-1A,dfrA14,fosA,oqxA,oqxB | 191 |
| k1490 | FLBJ01 | 15 | Human | Blood | United Kingdom | 2006 | aac(6')-Ib-cr,aph(6)-Id,blaCTX-M-15,blaOXA-1,blaSHV-106,blaTEM-1B,dfrA14,fosA,oqxA,oqxB | 191 |
| PO2673 | NFVU01 | 15 | Human | Blood | Nigeria | 9-Feb-12 | aac(3)-IIa,aac(6')-Ib-cr,aph(6)-Id,blaCTX-M-15,blaOXA-1,blaSHV-106,blaTEM-1B,dfrA14,fosA,oqxA,oqxB | 191 |
| C1699 | NXBL01 | 15 | Human | urine | Portugal: Oporto | 2012 | aac(6')-Ib-cr,aph(6)-Id,blaCTX-M-15,blaOXA-1,blaSHV-106,blaTEM-1B,dfrA14,fosA,oqxA,oqxB | 192 |
| H1119 | NXBK01 | 15 | Human | urine | Portugal: Oporto | 2010 | aac(3)-Ia,aadA1,aadA2,aph(3'')-Ib,aph(3')-Ia,aph(6)-Id,blaSHV-2,dfrA12,fosA,oqxA,oqxB,sul1 | 192 |
| AUH_KIMP195 | NIFL01 | 15 | Human | - | Lebanon | 20-May-13 | aac(3)-IIa,aac(6')-Ib-cr,aph(6)-Id,blaCTX-M-15,blaOXA-1,blaOXA-48,blaSHV-106,blaTEM-1B,dfrA14,fosA,oqxA,oqxB,tet(A) | 193 |
| EuSCAPE_HU042 | ULCK01 | 15 | Human | Urine | Hungary | 2013 | aac(3)-IIa,aac(6')-Ib-cr,aph(6)-Id,blaCTX-M-15,blaOXA-534,blaSHV-106,blaTEM-1B,dfrA14,fosA,oqxA,oqxB | 193 |
| EuSCAPE_PT049 | UIVV01 | 15 | Human | Urine | Portugal | 2014 | aph(6)-Id,blaCTX-M-15,blaSHV-106,blaTEM-1B,fosA,oqxA,oqxB | 193 |
| FDAARGOS_567 | RKJD01 | 15 | Human | - | - | - | aac(3)-IIa,aac(3)-IId,aph(3'')-Ib,aph(6)-Id,blaCTX-M-15,blaOXA-1,blaSHV-106,blaTEM-1B,catA1,fosA,oqxA,oqxB,tet(B) | 194 |
| k1264 | FLBL01 | 15 | Human | Blood | United Kingdom | 2006 | aph(3'')-Ib,aph(6)-Id,blaCTX-M-15,blaSHV-106,blaTEM-1B,dfrA14,fosA,oqxA,oqxB,sul2,tet(A) | 194 |
| AUH_KIMP196 | NIFM01 | 15 | Human | - | Lebanon | 1-Jun-13 | aac(3)-IIa,aph(6)-Id,blaCTX-M-15,blaOXA-1,blaOXA-48,blaSHV-106,blaTEM-1B,dfrA14,fosA,oqxA,oqxB | 195 |
| EuSCAPE_BE013 | UJNW01 | 15 | Human | Urine | Belgium | 2014 | blaCTX-M-15,blaSHV-106,fosA,oqxA,oqxB | 195 |
| EuSCAPE_HU007 | ULAD01 | 15 | Human | Urine | Hungary | 2013 | aac(6')-Ib-cr,aph(3'')-Ib,aph(6)-Id,blaCTX-M-15,blaOXA-1,blaSHV-106,blaTEM-1B,blaVIM-4,dfrA14,fosA,mph(E),msr(E),oqxA,oqxB,sul1,sul2 | 195 |
| MRY10_897 | BDLH01 | 15 | Human | - | Japan | 2010 | aac(3)-IIa,aac(6')-Ib-cr,blaCTX-M-15,blaOXA-1,blaSHV-106,dfrA14,fosA,oqxA,oqxB,tet(A) | 195 |
| EuSCAPE_HU010 | ULBB01 | 15 | Human | Lower Respiratory Tract Secrection | Hungary | 2013 | aac(3)-IIa,aac(6')-Ib3,aph(3'')-Ib,aph(6)-Id,blaCTX-M-15,blaOXA-1,blaSHV-106,blaTEM-1B,blaVIM-4,dfrA14,fosA,mph(E),msr(E),oqxA,oqxB,sul1,sul2 | 196 |
| EuSCAPE_HU025 | ULBW01 | 15 | Human | Wound Secretion | Hungary | 2013 | aac(6')-Ib,aph(3'')-Ib,aph(6)-Id,blaCTX-M-15,blaSHV-106,blaTEM-1B,blaVIM-4,dfrA14,fosA,mph(E),msr(E),oqxA,oqxB,sul1,sul2 | 196 |
| EuSCAPE_HU026 | ULBU01 | 15 | Human | Blood | Hungary | 2014 | aac(6')-Ib,aph(3'')-Ib,aph(6)-Id,blaCTX-M-15,blaSHV-106,blaTEM-1B,blaVIM-4,dfrA14,fosA,mph(E),msr(E),oqxA,oqxB,sul1,sul2 | 196 |
| EuSCAPE_PT073 | UIWS01 | 15 | Human | Urine | Portugal | 2014 | aac(6')-Ib11,aadA1,aph(3'')-Ib,aph(6)-Id,blaCTX-M-15,blaKPC-3,blaOXA-9,blaSHV-106,blaTEM-1B,dfrA14,fosA,oqxA,oqxB,sul2 | 196 |
| 3189STDY5864798 | FXRF01 | 15 | - | Peritoneal dialysis catheter | Pakistan | 2010/2012 | aac(6')-Ib,aph(3'')-Ib,aph(6)-Id,armA,blaCTX-M-15,blaSHV-106,blaVEB-5,dfrA14,fosA,mph(E),msr(E),oqxA,oqxB,sul1,sul2 | 197 |
| EuSCAPE_FR002 | UKCR01 | 15 | Human | Urine | France | 2013 | aph(3'')-Ib,aph(6)-Id,blaCTX-M-15,blaSHV-106,blaTEM-1B,fosA,oqxA,oqxB,sul2,tet(A) | 197 |
| EuSCAPE_HU028 | ULBZ01 | 15 | Human | Urine | Hungary | 2014 | aac(6')-Ib3,aph(3'')-Ib,aph(6)-Id,blaSHV-106,blaVIM-4,fosA,mph(E),msr(E),oqxA,oqxB,sul1 | 197 |
| Kp891 | QKNG01 | 15 | Human | rectal smear | Spain:Barcelona | 20-Feb-13 | aph(6)-Id,blaCTX-M-15,blaOXA-1,blaOXA-48,blaSHV-106,blaTEM-1B,fosA,oqxA,oqxB | 197 |
| EuSCAPE_ES079 | ULAL01 | 15 | Human | Wound Secretion | Spain | 2013 | aac(6')-Ib-cr,aph(3'')-Ib,aph(6)-Id,blaCTX-M-15,blaOXA-1,blaOXA-48,blaSHV-106,blaTEM-1B,dfrA14,fosA,oqxA,oqxB,qepA1,rmtB,sul2 | 198 |
| EuSCAPE_HU003 | ULCS01 | 15 | Human | Urine | Hungary | 2013 | aac(3)-IIa,aac(6')-Ib3,aph(3'')-Ib,aph(6)-Id,blaCTX-M-15,blaOXA-1,blaSHV-106,blaVIM-4,dfrA14,fosA,mph(E),msr(E),oqxA,oqxB,sul1 | 198 |
| 3189STDY5864856 | FXOE01 | 15 | Human | blood | Pakistan | 2010/2012 | aac(6')-Ib3,aadA1,aph(3'')-Ib,aph(6)-Id,armA,blaCTX-M-15,blaNDM-1,blaOXA-10,blaSHV-106,blaTEM-1B,blaVEB-5,dfrA1,fosA,oqxA,oqxB,sul1,sul2,tet(D) | 199 |
| EuSCAPE_ES039 | ULCU01 | 15 | Human | Blood | Spain | 2014 | aac(6')-Ib-cr,aph(3'')-Ib,aph(6)-Id,blaCTX-M-15,blaOXA-1,blaOXA-48,blaSHV-106,blaTEM-1B,dfrA14,fosA,oqxA,oqxB,qepA1,rmtB,sul2 | 199 |
| EuSCAPE_ES054 | UKZO01 | 15 | Human | Blood | Spain | 2013 | aac(6')-Ib-cr,aph(3'')-Ib,aph(6)-Id,blaCTX-M-15,blaOXA-1,blaOXA-48,blaSHV-106,blaTEM-1B,dfrA14,fosA,oqxA,oqxB,qepA1,rmtB,sul2 | 199 |
| EuSCAPE_ES078 | ULAK01 | 15 | Human | Urine | Spain | 2013 | aac(6')-Ib-cr,aph(3'')-Ib,aph(6)-Id,blaCTX-M-15,blaOXA-1,blaOXA-48,blaSHV-106,blaTEM-1B,dfrA14,fosA,oqxA,oqxB,qepA1,rmtB,sul2 | 199 |
| EuSCAPE_ES083 | ULAN01 | 15 | Human | Urine | Spain | 2013 | aac(6')-Ib-cr,aph(3'')-Ib,aph(6)-Id,blaCTX-M-15,blaOXA-1,blaOXA-48,blaSHV-106,blaTEM-1B,dfrA14,fosA,oqxA,oqxB,sul2 | 199 |
| EuSCAPE_HU011 | ULBE01 | 15 | Human | Urine | Hungary | 2013 | aac(3)-IIa,aac(6')-Ib3,aph(3'')-Ib,aph(6)-Id,blaCTX-M-15,blaOXA-1,blaSHV-106,blaTEM-1B,blaVIM-4,dfrA14,fosA,mph(E),msr(E),oqxA,oqxB,qnrB19,sul1,sul2 | 199 |
| EuSCAPE_TR175 | UJXX01 | 15 | Human | Urine | Turkey | 2013 | aph(3'')-Ib,aph(6)-Id,blaSHV-106,blaTEM-1B,fosA,oqxA,oqxB,sul2,tet(A) | 199 |
| ERS480609 | CCFX01 | 15 | - | - | - | - | aac(3)-IIa,aac(6')-Ib-cr,aph(3'')-Ib,aph(6)-Id,blaCTX-M-15,blaSHV-106,blaTEM-1B,dfrA14,fosA,oqxA,oqxB,sul2,tet(A) | 200 |
| EuSCAPE_ES055 | UKZP01 | 15 | Human | Wound Secretion | Spain | 2013 | aac(6')-Ib-cr,aph(3'')-Ib,aph(6)-Id,blaCTX-M-15,blaOXA-1,blaOXA-48,blaSHV-106,blaTEM-1B,dfrA14,fosA,oqxA,oqxB,qepA1,rmtB,sul2 | 200 |
| EuSCAPE_ES062 | ULAI01 | 15 | Human | Wound Secretion | Spain | 2014 | aac(6')-Ib-cr,aph(3'')-Ib,aph(6)-Id,blaCTX-M-15,blaOXA-1,blaOXA-48,blaSHV-106,blaTEM-1B,dfrA14,fosA,oqxA,oqxB,sul2 | 200 |
| EuSCAPE_HU005 | UKZN01 | 15 | Human | Wound Secretion | Hungary | 2013 | aac(6')-Ib-cr,aph(3'')-Ib,aph(6)-Id,blaCTX-M-15,blaOXA-1,blaSHV-106,blaTEM-1B,blaVIM-4,dfrA14,fosA,mph(E),msr(E),oqxA,oqxB,sul1,sul2 | 200 |
| EuSCAPE_HU006 | UKZY01 | 15 | Human | Urine | Hungary | 2013 | aac(6')-Ib,aph(3'')-Ib,aph(6)-Id,blaCTX-M-15,blaSHV-106,blaTEM-1B,blaVIM-4,fosA,mph(E),msr(E),oqxA,oqxB,sul1,sul2 | 200 |
| EuSCAPE_HU008 | ULAS01 | 15 | Human | Urine | Hungary | 2013 | aac(3)-IIa,aac(6')-Ib3,aph(3'')-Ib,aph(6)-Id,blaCTX-M-15,blaOXA-1,blaSHV-106,blaTEM-1B,blaVIM-4,dfrA14,fosA,mph(E),msr(E),oqxA,oqxB,sul1,sul2 | 200 |
| EuSCAPE_HU036 | ULCC01 | 15 | Human | Urine | Hungary | 2013 | aac(3)-IIa,aac(6')-Ib3,aph(3'')-Ib,aph(6)-Id,blaCTX-M-15,blaOXA-1,blaSHV-106,blaTEM-1B,blaVIM-4,dfrA14,fosA,mph(E),msr(E),oqxA,oqxB,sul1,sul2 | 200 |
| k2329 | FLGG01 | 15 | Human | bacterial isolates collected | - | 2010 | aac(3)-IIa,aac(6')-Ib-cr,blaCTX-M-15,blaOXA-1,blaSHV-106,catA1,dfrA14,fosA,oqxA,oqxB,qnrB1 | 200 |
