# Supplementary material for: Whole genome sequencing of OXA-232-producing wzi93-KL112-O1 carbapenem-resistant Klebsiella pneumoniae in human bloodstream infection co-harboring chromosomal ISEcp1-based bla CTX-M-15 and one rmpA2-associated virulence plasmid
Source: Front Cell Infect Microbiol. 2022 Sep 29;12:984479. doi: 10.3389/fcimb.2022.984479 (PMC9560801; doi:10.3389/fcimb.2022.984479)
Supplement: Supplementary file 3 [file Table_2.docx]

**Table S2. Genbank accession numbers of all 45 OXA-232 producing *K. pneumoniae* strains.**

| **Strains name** | **Genbank accession numbers** |
| --- | --- |
| KPTCM | CP097385 |
| SHK051 | SIZO01 |
| SHK128 | SIZT01 |
| SHK140 | SIZU01 |
| SHK022 | SIZL01 |
| SHK032 | SIZM01 |
| SHK038 | SIZN01 |
| WSD411 | CP045674 |
| SHK059 | SIZP01 |
| SHK072 | SIZQ01 |
| SHK096 | SIZR01 |
| G412_2 | QRGJ01 |
| G415_1 | QRGI01 |
| H401_2 | QRGH01 |
| E104_1 | QRGM01 |
| G407_2 | QRGK01 |
| H420_4 | QRGE01 |
| SHK012 | SIZK01 |
| E109_1 | QRGN01 |
| E111_1 | QRGL01 |
| H415_1 | QRGG01 |
| H415_2 | QRGF01 |
| SHK103 | SIZS01 |
| HD6655 | JABMLS010000001 |
| HD6665 | JABMLR010000001 |
| HD6703 | JABMLQ010000001 |
| EN5275 | VINI01000001 |
| NB5306 | QYCO01000001 |
| KL8 | REGZ01000001 |
| KL29 | REHA01000001 |
| EN5199 | VSLC00000000 |
| EN5338 | JAAGUA000000000 |
| Kp06 | NTFP00000000 |
| KP41-2015 | MCNI00000000 |
| BA24831 | JACWMO000000000 |
| PM565 | MNPB00000000 |
| PM1842 | MNPC00000000 |
| PM138 | MNPG00000000 |
| B1647 | MCFO00000000 |
| 80CAM | JAAEYE000000000 |
| KPN601 | SJKQ00000000 |
| KPN602 | SJKP00000000 |
| KPN605 | SJKV00000000 |
| KPN606 | SJKN00000000 |
| KPN612 | SJKU00000000 |
